# Validation study of risk prediction models for female relatives of Australian women with breast cancer

**DOI:** 10.1186/1897-4287-10-S2-A66

**Published:** 2012-04-12

**Authors:** R MacInnis, G Dite, A Bickerstaffe, J Dowty, K Aujard, C Apicella, K Phillips, P Weideman, J Hopper

**Affiliations:** 1 Centre for MEGA Epidemiology, University of Melbourne, Australia; 2 Cancer Epidemiology Centre, The Cancer Council Victoria, Australia; 3 Division of Haematology & Medical Oncology, Peter MacCallum Cancer Centre, Australia

## 

Risk prediction algorithms are an important tool for identifying individuals at high risk of developing the disease who can then be offered individually tailored clinical management. Several algorithms that predict the probability of breast cancer incidence are currently used in clinical practice. It is uncertain, though, as to which of the breast cancer risk prediction models performs best for female relatives of Australian women with breast cancer.

We evaluated the performance of the risk prediction algorithms BOADICEA, BRCAPRO and the Gail model using 879 families of ABCFS case probands, half of whom were diagnosed before age 40 years and the remainder before age 60 years. Cumulative breast cancer risks over 10 years of follow-up were calculated for 2000 unaffected female relatives.

A total of 93 incident breast cancers were reported. The ratios (95% CI) of expected to observed number of breast cancers were 0.69 (0.56-0.84) using BOADICEA, 0.62 (0.51-0.77) using BRCAPRO, and 0.89 (0.73-1.09) using the Gail model. Tests for discrimination (ROC curve) were BOADICEA=0.61, BRCAPRO=0.64, and the Gail model=0.65.

Similar analyses using other models, including the Tyrer-Cuzick (IBIS) and Claus models, will also be presented.

